# Transactions of the Semi-Annual Meeting of the Medical Society of Erie Co., N. Y.

**Published:** 1855-07

**Authors:** 


*Transactions of the Semi-annual Meeting of the Medical Society of Erie Co., N. Y.*—The semi-annual meeting of the Medical Society of the county of Erie, was held in the city at American Hall, June 12, 1855.

The society was called to order at 11¼ o'clock, A. M., by the president, Prof. James P. White. Forty-one members were in attendance during the session.

The minutes of the annual meeting in January last, and of a special meeting in February, were read by the secretary, and severally adopted.

The following gentlemen were, upon their applications, and after an examination of their credentials, elected to membership with the society:—Drs. Julius T. Minor, Jeremiah N. Brown, P. P. E. Tobie, and George Abbott.

*The committee to confer with the German medical practitioners*, appointed in January last, made a verbal *report* through Dr. Stork, stating that no action was needed at this time by the members of the German society which had been recently organized, as they should take means to legally qualify themselves for membership with the Erie Co. society, except in the case of Dr. Devening, who had an American diploma, and who had once been in membership with this society, and again wished to become a member.

On *motion* of Dr. Hunt, the *report* was accepted, the committee discharged, and a committee of three appointed to whom the application of Dr. Devening was referred for an examination into the circumstances connected with his previous membership with the society.

Drs. Hunt, Nelson, and Wyckoff, were appointed on said committee, and subsequently, on the motion of Dr. Nelson, the president was added.

The *oration* was delivered by Dr. E. P. Smith. Subject: "*The application of the Microscope to the Diagnosis of Disease.*"

At its conclusion the thanks of the society were, on *motion* of Dr. Boardman, voted the speaker for his interesting address, and a copy requested for preservation among the papers of the society.

Dr. Nelson offered the following resolution:

*Resolved*, That in future no applications for membership be received without a certificate from the county clerk accompanying the credentials.

The chair ruled that this resolution partook of the nature of an alteration of the By-laws, and as such the society could not act upon it without the requisite notice.

The resolution was then submitted as an amendment to lie over until the next regular meeting.

Dr. Baker was appointed *orator* for the next meeting, and Dr. Hutchins his *substitute.*

On *motion* of Dr. Rochester, it was

*Resolved*, That a committee of three be appointed to make arrangements for a society dinner at the next January session.

Drs. Rochester, Samo, and Van Deventer, were appointed such committee.

At this stage of the proceedings, Dr. Hutchins inquired if Dr. Ring had resigned the medical appointment from the county authorities, in accordance with his promise made to this society in October last at the special meeting.

In the absence of any answer to the inquiry, Dr. Hutchins

*Moved*, That the secretary ascertain from Dr. Ring whether he now holds the office of physician to the city poor under an appointment of the Superintendents of the Poor, at a less compensation than that fixed in the bill of prices established by this society.

The resolution was *adopted.*

Dr. Nelson

*Moved*, That the secretary propound similar questions to all members holding office under city or county appointments.

*Adopted.*

Dr. Abbott inquired whether the society had a tariff of prices regulating charges for medical and surgical services in the country, and remarked upon its great convenience, and urged, if none existed, the propriety of taking some action in the matter.

There is no such fee bill, but no action was taken relative to establishing one at this time.

Dr. Baker offered the following:

*Resolved*, That Dr. E. P. Smith be requested to continue his microscopic investigations and report to the society at its next meeting.

*Adopted.*

The society proceeded to the consideration of the amendments of the By-laws proposed at the January meeting.

The vote being taken upon the amendments offered by Dr. Hamilton, the first amendment, that of the Title, striking out the words "*Rules and Regulations*" from the same, was, upon motion of Dr. Nelson, *adopted.*

The amendment to Article Second, substituting the word "delegates" for "delegate," was also, on motion of Dr. Nelson, *adopted.*

The amendment to the "Tariff of Prices," proposed by Dr. Wilcox, fixing the salary of the Health Physician at nine hundred dollars per year, was, on motion of Dr. Whitney, *adopted.*

After which the society *adjourned.* J. M. N.

